# Hormonal contraceptives and the risk of meningioma: A Swedish register-based case-control study

**DOI:** 10.1093/neuonc/noaf228

**Published:** 2025-10-07

**Authors:** Giorgio Tettamanti, Xiaochen Shu, Hanna Mogensen, Helena Kopp Kallner, Tiit Mathiesen, Maria Feychting

**Affiliations:** Institute of Environmental Medicine, Karolinska Institutet, Stockholm, Sweden (G.T., H.M., M.F.); Department of Epidemiology, School of Public Health, Suzhou Medical College of Soochow University, Suzhou, China (X.S.); Institute of Environmental Medicine, Karolinska Institutet, Stockholm, Sweden (G.T., H.M., M.F.); Department of Immunology, Uppsala University, Uppsala, Sweden (H.M.); Department of Clinical Sciences, Karolinska Institutet, Stockholm, Sweden (H.K.K.); Department of Obstetrics and Gynecology, Danderyds Hospital, Danderyd, Sweden (H.K.K.); Department of Neurosurgery, Rigshospitalet, Copenhagen, Denmark (T.M.); Department of Clinical Medicine, University of Copenhagen, Copenhagen, Denmark (T.M.); Department of Clinical Neuroscience, Karolinska Institutet, Stockholm, Sweden (T.M.); Institute of Environmental Medicine, Karolinska Institutet, Stockholm, Sweden (G.T., H.M., M.F.)

## Abstract

**Background:**

Studies of hormonal contraceptives and the risk of meningioma has often relied on self-reported information, primarily focusing on oral contraceptives. Recently, an association between progestins and meningioma was suggested. We aimed to analyze the association between hormonal contraceptives and meningioma using data from Swedish national registries.

**Methods:**

We used the Swedish Cancer Register to identify women born 1955-1995, diagnosed with meningioma at age ≥20 years, between 2007 and 2015 (*N *= 1055). Twenty controls per case were randomly selected from the Swedish population register, matched by birth year and county of residence. We retrieved information regarding prescriptions of hormonal contraceptives from the National Prescribed Drug Register. Adjusted conditional logistic regression models were used to evaluate the association between hormonal contraceptive use and meningioma occurrence.

**Results:**

Women prescribed hormonal contraceptives ≥1 year before the index date had an odds ratio (OR) of meningioma of 1.76 (95% CI, 1.53-2.03). For contraceptives containing medroxyprogesterone the OR was 5.49 (95% CI, 4.51-6.67), while the association was weaker for other progesterone contraceptives (OR = 1.34, 95% CI, 1.09-1.65). No association was found for intrauterine devices, vaginal rings, and subdermal implants.

**Conclusions:**

This large register-based case-control study show a strong association between injectable hormonal contraceptives containing medroxyprogesterone and meningioma risk. The results add to the growing body of evidence of an association between meningiomas and progestins in general and the strong, consistent associations suggest a causal role of injectable medroxyprogesterone in meningioma growth. Our results correspond to 2 additional cases of meningioma per 10 000 women exposed to medroxyprogesterone per year.

Key Points

- Meningioma risk was increased in women prescribed medroxyprogesterone injections
- A weak association between other progesterone contraceptives and meningioma was found
- No association was found for intrauterine devices, vaginal rings, and subdermal implants

Importance of the study

An association between the use of hormonal contraceptives and the risk of meningioma has been suggested, but available evidence is either based on crude exposure information with few details about specific types of hormonal contraceptives, or few exposed women. We present a large nationwide registry-based study, with detailed information about prescriptions of different hormonal contraceptives, linked to the population-based cancer register. Women who were prescribed medroxyprogesterone injections had a 5-fold increased odds of meningioma. For other progesterone contraceptives a weak association with meningioma was observed. No association was found for prescriptions of intrauterine devices, vaginal rings, and subdermal implants. The robustness of the findings was evaluated in sensitivity analyses, which all confirmed the original results. The findings correspond to 2 additional cases of meningioma per 10 000 women exposed to medroxyprogesterone injections per year.

Meningiomas account for 20%-42% of all intracranial tumors, with an incidence ranging between 2 and 8 per 100 000.^1-3^ Established risk factors for meningiomas are increasing age, female sex, exposure to ionizing radiation, high BMI, and some rare genetic syndromes, such as neurofibromatosis type 1 and type 2, Turner’s syndrome, and Werner’s syndrome.^1^^,^^4^

The increased incidence of meningioma observed in women has fueled hypotheses of a causal link between female sex hormones and the development of meningioma.^1^^,^^5-8^ A biological mechanism has been suggested to involve receptors for sex-hormones. Estrogen and androgen receptor expression is low and inconsistent, but progesterone receptor expression is found in 70%-90% of all meningiomas, inversely related to degree of aggressiveness.^9-11^ In addition, progesterone receptor expression is higher in meningiomas that developed during hormonal treatment than controls: 89% vs 71%.^9^ Yet, epidemiological studies on the association between exposure to female sex hormones and a diagnosis of meningioma have been contradictory; some have reported an increased risk of meningioma with the use of oral contraceptives,^12-15^ while others found no association.^6^^,^^16-18^ Surprisingly, pregnancy, with very high levels of endogenous sex hormones, even appeared to protect from meningiomas.^19^ Recently, however, 2 studies suggested progestin treatment as a risk factor for meningiomas in French and US populations,^20^^,^^21^ and the Pharmacovigilance Risk Assessment Committee of the European Medicines Agency issued documents in November of 2024 stating, “Cases of meningioma (single and multiple) have been reported in patients treated with medroxyprogesterone acetate for a prolonged time.”^22^

Use of contraceptives and progestins varies between countries in terms of indications and preferred medications.^23^ There is a need for data from comprehensive population-based tumor registries along with objective information of hormonal contraception for traceable epidemiological observations on the association between hormonal contraception use and meningioma diagnoses.

The aim of the current study is to evaluate the association of treatment with hormonal contraceptives and the risk of meningioma among young and middle-aged women using data from nationwide Swedish healthcare registers, including the National Prescribed Drug Register.

## Methods

### Study population

The study was designed as a register-based case-control study nested within the Swedish population. Linkages between the population register and health data registers were made available through the unique personal identification number assigned to all Swedish inhabitants.^24^ Eligible for inclusion were women aged 20 years or older who were born between 1955 and 1995, with no previous central nervous system tumor reported to the Swedish National Cancer Register at start of follow-up. Incident meningioma cases were identified through the Cancer Register between January 1, 2007 and December 31, 2015. The Swedish National Cancer Register contains information on all malignant tumors as well as certain benign tumors (including benign brain tumors) diagnosed in Sweden since 1958.^25^ Meningioma cases were identified using the third revision of the International Classification of Disease for Oncology (ICD-O-3 C70, morphology code starting with 953). For each meningioma case, twenty controls were randomly selected among eligible women living in Sweden at the time of diagnosis of the case (incidence density sampling), individually matched to the case by birth year and county of residence at index date (date of meningioma diagnosis). As an additional eligibility criteria, cases and controls were included in the study only if they had been living in Sweden at least 3 years before the index date.

### Exposure

Hormonal contraceptives are used to prevent unwanted pregnancies, in Sweden usually prescribed by midwifes, but they can also be prescribed for other indications, such as treatment of acne, heavy menstrual bleeding, dysmenorrhea, or premenstrual symptoms. All prescribed contraceptives are free of charge up to age 18 years, thereafter, subsidized until age 25 years, and from 25 years of age, women pay prescribed contraceptives in full.

We obtained information about prescriptions of hormonal contraceptives from the National Prescribed Drug Register, which contains nationwide information on all dispensed medications since July 2005, including Anatomical Therapeutic Chemical (ATC) codes and mode of delivery.^26^ The register does not include information about indications for the prescription. Hormonal contraceptives prescriptions were identified using the ATC codes G03A, G02BA03, and G02BB and were further classified as progestin only contraception (ATC codes G03AC, G02BA03, and G02BB02), medroxyprogesterone (ATC code G03AC06), combined hormonal contraception (estrogens and progestogens in combination, ATC codes G03AA, G03AB, and G02BB01), and combined hormonal contraception with levonorgestrel (ATC codes G03AA07 and G03AB03). In addition, we also identified women who received prescriptions of intrauterine devices containing levonorgestrel (ATC code G02BA03), subdermal implants (based on information on mode of delivery), and vaginal rings (ATC code G02BB), also combined contraceptives.

In the main analysis, women were defined as exposed if they had at least one prescription of a hormonal contraceptive at least 1 year before the index date. We evaluated the association between prescriptions of any type of hormonal contraceptives and meningioma occurrence, as well as specific types of hormonal contraceptives separately. In sensitivity analyses we evaluated 2 longer induction periods: at least one prescription 2 years before the index date, and at least one prescription 3 years before the index date. In addition, we conducted 2 sensitivity analyses to reduce exposure misclassification from dispensed prescriptions that were never used by requiring 2 or more prescriptions at least 2 years before the index date in one analysis, and 2 or more prescriptions at least 3 years before the index date in the other. The reference group in all analyses were women who did not have any prescription of hormonal contraceptives in the National Prescribed Drug Register prior to index date. In the sensitivity analyses in which the hormonal contraceptives had to be prescribed at least 2 (or 3 years) before the index date, only meningioma cases who were diagnosed from 2008 (or 2009) were included.

As registration of a cancer diagnosis can be delayed in the Swedish Cancer Register, especially for benign tumors, a sensitivity analysis was conducted where the date of meningioma diagnosis was defined using the earliest date reported in either the Swedish Cancer Register or the National Patient Register. The latter contains nationwide information on inpatient care since 1987, and outpatient care since 2001.^27^ To assess potential confounding from conditions that may influence the likelihood of being prescribed hormones or a specific type of hormone, we conducted sensitivity analyses excluding women born with congenital malformations or women who had any previous cancer diagnosis. We also performed age-stratified analyses (<45 years/≥45 years). In a subset of women, we further adjusted for body mass index using information on height and weight obtained from the National Medical Birth Register (MBR), which contains information on antenatal, obstetrical, and neonatal care for approximately 98% of pregnancies resulting in a live birth.^28^ The most recent record in the MBR that predated the index date was used to obtain information on height and weight during the first antenatal visit: this information is available from 1982. This analysis was restricted to women who had given birth 1982 or later.

We made a post hoc decision to further assess the robustness of the findings by creating a separate case-control study with the additional eligibility criteria that participants should have no contraindications for hormonal contraceptives prescription before the index date and made a new random selection of controls using incidence density sampling. These contraindications were defined according to the WHO Medical eligibility criteria for contraceptive use, fourth edition (Table S1).^29^ In a post hoc analysis, diagnoses reported to the National Patient Register were used to determine whether women who were prescribed hormonal contraceptives had a different medical history compared to women who had no prescriptions, in order to evaluate potential confounding by indication.

### Statistical analysis

Conditional logistic regression models were used to estimate the association between hormonal contraceptives and the risk of meningioma. Analyses were adjusted for marital status (married/not married), income (categorized in quartiles based on the yearly income distribution in the whole female Swedish population), educational level (compulsory education or less, upper secondary education, postsecondary education), parity (no children, 1 or 2 children, 3 children or more), country of birth (born in Sweden/not born in Sweden), history of diseases of the circulatory system, family history of breast cancer and family history of central nervous system tumors. Information regarding marital status, income, and education was obtained from the Longitudinal Integration Database for Health Insurance and Labour Market Studies (LISA),^30^ parity from the Multi-generation Register,^31^ country of birth from the Total Population Register,^32^ history of diseases of the circulatory system from the National Patient Register,^27^ and family history of breast cancer and central nervous system tumor was obtained using the Multi-generation Register, to identify first-degree relatives, and the Swedish National Cancer Register. We estimated the excess number of meningioma cases due to medroxyprogesterone exposure by calculating the attributable fraction, according to Rothman et al,^33^ under the assumption that the observed association is causal. From the Prescribed Drug register we estimated the number of women born 1955-1995 who had ever been prescribed medroxyprogesterone in the study population during the study period, in total 88 513 women. All statistical analyses have been performed using SAS statistical software version 9.4 (SAS Institute, Inc, Cary, NC, USA). The study has been approved by the Regional Ethical Review Board in Stockholm (2011/634-31/4, 2016/27-32, 2024-06399-02), that also waived the requirement for participant consent.

## Results

Between 2007 and 2015, 1055 women born between 1955 and 1995 had a diagnosis of a meningioma reported to the Swedish Cancer Register after age 20. Descriptive characteristics of the 1055 cases and 21 100 matched controls are reported in Table 1. The large majority of the meningioma cases (82%) was forty years or older at the time of diagnosis and no or small differences were observed between cases and controls with regards to parity, marital status, income, country of birth, history of circulatory diseases, and family history of breast cancer and central nervous system tumors: however, controls were slightly more likely to have completed postsecondary education (41% vs. 36%). Table S2 shows the distribution of prescribed hormonal contraceptives among the controls according to descriptive characteristics.

**Table 1.** Descriptive characteristics of the included meningioma cases and matched controls

	Meningioma cases (*n *= 1055)	Matched controls (*n *= 21 100)	*P*-value
**Age at index date**			
20-29	29 (3%)	580 (3%)	1.0
30-39	156 (15%)	3120 (15%)	
40-49	478 (45%)	9560 (45%)	
50-60	392 (37%)	7840 (37%)	
**Parity**			
No children	167 (16%)	3437 (16%)	.925
One or two children	591 (56%)	11753 (56%)	
Three children or more	297 (28%)	5910 (28%)	
**Marital status**			
Not married	535 (51%)	10479 (50%)	.507
Married	520 (49%)	10621 (50%)	
**Educational level**			
Compulsory or less	126 (12%)	2292 (11%)	.028
Upper secondary education	539 (51%)	10022 (48%)	
Postsecondary education	385 (36%)	8679 (41%)	
Missing	5 (0%)	107 (1%)	
**Income (percentiles)**			
<25th	242 (23%)	4688 (22%)	.408
25th-50th	255 (24%)	4895 (23%)	
50th-75th	290 (27%)	5611 (27%)	
75th-100th	268 (25%)	5891 (28%)	
Missing	-	15 (0%)	
**Born in Sweden**	879 (83%)	17331 (82%)	.328
**History of diseases of the circulatory system**	99 (9%)	1437 (7%)	.001
**Family history of breast cancer**	71 (7%)	1494 (7%)	.664
**Family history of central nervous system tumors**	22 (2%)	383 (2%)	.523

Forty-three percent of the meningioma cases were prescribed hormonal contraceptives at least 1 year before the date of diagnosis, compared to 32% of the controls (Table 2). An increased odds ratio (OR) of meningioma was found among women who were prescribed any hormonal contraceptive prescriptions at least 1 year before the index date (OR = 1.76, 95% CI, 1.53-2.03). A slightly stronger association was observed for progestin only contraceptives (OR = 1.92, 95% CI, 1.66-2.21), while a 5-fold increased odds of meningioma was found among women who were prescribed hormonal contraceptives containing medroxyprogesterone (OR = 5.49, 95% CI, 4.51-6.67). For progestin only contraceptives that did not contain medroxyprogesterone, only a weak association was observed (OR = 1.19, 95% CI, 0.99-1.41). A modest increased OR of meningioma was observed for contraceptives containing estrogens and progestogens or estrogens and levonorgestrel in combination, while subdermal implants, intrauterine devices, and vaginal rings were not associated with meningioma occurrence (Table 2). Similar findings were found in age-stratified analyses (Table 2) and in analyses that were further adjusted for body mass index (Table S3).

**Table 2.** Association between hormonal contraceptives, prescribed at least 1 year before the index date, and the occurrence of meningioma

			Age at index date < 45		Age at index date ≥ 45	
	*N* cases/*N* controls	**Adjusted OR** ^a^ **(95% CI)**	*N* cases/*N* controls	**Adjusted OR** ^a^ **(95% CI)**	*N* cases/*N* controls	**Adjusted OR** ^a^ **(95% CI)**
**Any hormonal contraceptive**						
Unexposed^b^	573/13741	Ref	149/3718	ref	424/10023	ref
Exposed	456/6851	1.76 (1.53-2.03)	224/3734	1.57 (1.25-1.97)	232/3117	1.89 (1.58-2.25)
**Progestin only contraceptive**						
Unexposed^b^	573/13741	Ref	149/3718	ref	424/10023	ref
Exposed	402/5503	1.92 (1.66-2.21)	182/2735	1.73 (1.36-2.20)	220/2768	2.02 (1.69-2.41)
**Medroxyprogesterone**						
Unexposed^b^	573/13741	ref	149/3718	ref	424/10023	ref
Exposed	186/853	5.49 (4.51-6.67)	72/336	5.55 (3.92-7.85)	114/517	5.44 (4.28-6.90)
**Progestins excluding medroxyprogesterone**						
Unexposed^b^	573/13741	ref	149/3718	ref	424/10023	ref
Exposed	216/4637	1.19 (0.99-1.41)	110/2388	1.19 (0.90-1.56)	106/2249	1.17 (0.93-1.47)
**Estrogens and progestogens**						
Unexposed^b^	573/13741	Ref	149/3718	ref	424/10023	ref
Exposed	120/2345	1.22 (0.96-1.56)	93/1727	1.31 (0.96-1.79)	27/618	1.06 (0.70-1.61)
**Estrogens and levonorgestrel**						
Unexposed^b^	573/13741	Ref	149/3718	ref	424/10023	ref
Exposed	60/1170	1.27 (0.93-1.75)	49/837	1.51 (1.03-2.22)	11/333	0.84 (0.45-1.59)
**Subdermal implants**						
Unexposed^b^	573/13741	Ref	^c^	-	^c^	-
Exposed	10/247	1.04 (0.52-2.07)				
**Intrauterine devices and vaginal rings**						
Unexposed^b^	573/13741	Ref	149/3718	ref	424/10023	ref
Exposed	105/2527	1.01 (0.80-1.27)	47/1222	0.86 (0.60-1.25)	58/1305	1.11 (0.82-1.50)

a Adjusted for marital status, educational level, income, parity, history of diseases of the circulatory system, and family history of breast cancer and central nervous system tumors.

b The reference group in all analyses were women who did not have any prescription of hormonal contraceptives prior to index date.

c Less than 5 observations in one subgroup.

Results from sensitivity analyses in which different exposure definitions were used agree with the main findings (Table 3): when the exposure definition required at least 2 prescriptions of medroxyprogesterone the OR was 5.86, 95% CI, 4.75-7.22 for prescriptions at least 2 years before the index date and OR = 5.93, 95% CI, 4.76-7.39 for prescriptions at least 3 years before the index date. Excluding women with any previous cancer diagnosis, born with congenital malformations, or defining date of meningioma diagnosis as the earliest date reported in the Swedish Cancer Register or the National Patient Register, had a limited effect on the results (Table 4).

**Table 3.** Sensitivity analyses to evaluate the association between hormonal contraceptives and meningioma occurrence using different exposure definitions

	At least 1 prescription 2 years before the index date		At least 1 prescription 3 years before the index date		At least 2 prescriptions 2 years before the index date		At least 2 prescriptions 3 years before the index date	
	*N* cases/*N *controls	**Adjusted OR** ^a^ **(95% CI)**	*N* cases/*N* control	**Adjusted OR** ^a^ **(95% CI)**	*N* cases/*N* controls	**Adjusted OR** ^a^ **(95% CI)**	*N* cases/*N* controls	**Adjusted OR** ^a^ **(95% CI)**
**Any hormonal contraceptive**								
Unexposed^b^	533/12537	ref	480/11354	Ref	533/12537	ref	480/11354	ref
Exposed	401/6142	1.68 (1.45-1.95)	360/5400	1.74 (1.49-2.04)	319/4119	2.05 (1.75-2.40)	276/3320	2.14 (1.81-2.53)
**Progestins only**								
Unexposed^b^	533/12537	ref	480/11354	Ref	533/12537	ref	480/11354	ref
Exposed	350/4838	1.86 (1.60-2.16)	311/4156	1.94 (1.65-2.28)	262/2805	2.40 (2.03-2.83)	228/2373	2.49 (2.09-2.97)
**Medroxyprogesterone**								
Unexposed^b^	533/12537	ref	480/11354	Ref	533/12537	ref	480/11354	ref
Exposed	167/784	5.40 (4.40-6.63)	151/703	5.48 (4.42-6.80)	163/711	5.86 (4.75-7.22)	147/636	5.93 (4.76-7.39)
**Progestins excluding medroxyprogesterone**								
Unexposed^b^	533/12537	ref	480/11354	Ref	533/12537	Ref	480/11354	ref
Exposed	181/4037	1.12 (0.92-1.35)	157/3432	1.15 (0.94-1.41)	95/2059	1.15 (0.91-1.47)	78/1707	1.15 (0.88-1.49)
**Estrogens and progestogens**								
Unexposed^b^	533/12537	ref	480/11354	Ref	533/12537	ref	480/11354	ref
Exposed	104/2137	1.11 (0.86-1.44)	94/1908	1.13 (0.86-1.48)	77/1678	1.06 (0.79-1.41)	70/1467	1.10 (0.81-1.49)
**Estrogens and levonorgestrel**								
Unexposed^b^	533/12537	ref	480/11354	ref	533/12537	ref	480/11354	Ref
Exposed	50/1054	1.13 (0.80-1.59)	45/944	1.13 (0.79-1.62)	34/771	1.06 (0.71-1.58)	29/676	1.04 (0.67-1.59)
**Progestins excluding medroxyprogesterone**								
Unexposed^b^	533/12537	ref	480/11354	Ref	533/12537	Ref	480/11354	ref
Exposed	181/4037	1.12 (0.92-1.35)	157/3432	1.15 (0.94-1.41)	95/2059	1.15 (0.91-1.47)	78/1707	1.15 (0.88-1.49)
**Subdermal implants**								
Unexposed^b^	533/12537	ref	480/11354	ref	^c^	-	^c^	-
Exposed	9/211	1.05 (0.51-2.16)	9/181	1.20 (0.58-2.49)				
**Intrauterine devices and vaginal rings**								
Unexposed^b^	533/12537	Ref	480/11354	Ref	533/12537	Ref	^c^	-
Exposed	91/2150	1.02 (0.79-1.30)	78/1754	1.07 (0.82-1.40)	10/315	0.72 (0.37-1.38)		

a Adjusted for marital status, educational level, income, parity, history of diseases of the circulatory system, and family history of breast cancer and central nervous system tumors.

b The reference group in all analyses were women who did not have any prescription of hormonal contraceptives prior to index date.

c Less than 5 observations in one subgroup.

**Table 4.** Sensitivity analyses using the earliest date of meningioma diagnosis reported in the National Patient Register and the Swedish National Cancer Register and after removing women born with congenital malformations and women with any previous cancer diagnosis

	Date of diagnosis as the earliest date reported in either the National Patient Register or the Swedish Cancer Register		Excluding women who had a previous cancer diagnosis		Excluding women born with congenital malformations	
	*N* cases/*N* controls	**Adjusted OR** ^a^ **(95% CI)**	*N* cases/*N* controls	**Adjusted OR** ^a^ **(95% CI)**	*N* cases/*N* controls	**Adjusted OR** ^a^ **(95% CI)**
**Any hormonal contraceptive**						
Unexposed^b^	564/13487	Ref	504/12533	ref	535/13162	ref
Exposed	452/6828	1.73 (1.51-2.00)	414/6349	1.77 (1.53-2.05)	421/6532	1.79 (1.55-2.07)
**Progestins only**						
Unexposed^b^	564/13487	Ref	504/12533	Ref	535/13162	ref
Exposed	399/5502	1.88 (1.62-2.18)	365/5107	1.92 (1.65-2.23)	371/5246	1.93 (1.66-2.24)
**Medroxyprogesterone**						
Unexposed^b^	564/13487	Ref	504/12533	ref	535/13162	ref
Exposed	185/811	5.39 (4.43-6.57)	170/784	5.63 (4.57-6.93)	173/796	5.60 (4.57-6.87)
**Estrogens and progestogens**						
Unexposed^b^	564/13487	Ref	504/12533	ref	535/13162	ref
Exposed	118/2398	1.10 (0.87-1.40)	108/2175	1.21 (0.94-1.56)	110/2234	1.29 (1.00-1.67)
**Estrogens and levonorgestrel**						
Unexposed^b^	564/13487	ref	504/12533	ref	535/13162	ref
Exposed	59/1251	1.05 (0.77-1.44)	53/1075	1.29 (0.92-1.82)	53/1117	1.29 (0.92-1.81)
**Progestogens without Medroxyprogesterone**						
Unexposed^b^	564/13487	Ref	504/12533	ref	535/13162	ref
Exposed	214/4686	1.11 (0.93-1.32)	195/4311	1.18 (0.98-1.42)	198/4438	1.18 (0.98-1.41)
**Subdermal implants**						
Unexposed^b^	564/13487	ref	504/12533	ref	535/13162	ref
Exposed	10/222	1.22 (0.59-2.50)	9/232	1.04 (0.50-2.14)	9/233	0.97 (0.47-2.02)
**Intrauterine devices and vaginal rings**						
Unexposed^b^	564/13487	ref	504/12533	ref	535/13162	ref
Exposed	103/2602	0.95 (0.75-1.20)	93/2350	0.97 (0.76-1.24)	100/2426	1.01 (0.79-1.28)

a Adjusted for marital status, educational level, income, parity, history of disease of the circulatory system, and family history of breast cancer and central nervous system tumors.

b The reference group in all analyses were women who did not have any prescription of hormonal contraceptives prior to index date.

In post hoc analyses, we examined whether women who had prescriptions of hormonal contraceptives had a different medical history than others. Women who were prescribed medroxyprogesterone contraceptives were more likely to have certain comorbidities, such as endocrine and metabolic diseases, mental, behavioral and neurodevelopmental disorders, diseases of the nervous, digestive and musculoskeletal system, and congenital malformations and chromosomal abnormalities (Table S4). Analyses based on a new case-control study restricted to women who had no contraindications for hormonal contraceptives prescription before the index date were eligible for inclusion as cases or controls, provided results very similar to the main findings (Table S5).

The attributable fraction for medroxyprogesterone exposure was estimated at 81.8%, ≈ 152 cases among the exposed over the 9-year study period. This corresponds to approximately 2 excess cases of meningioma per 10 000 exposed women per year.

## Discussion

In this large nationwide register-based case-control study we observed a higher odds of meningioma in young and middle-aged women who were prescribed hormonal contraceptives. This association was almost entirely explained by medroxyprogesterone contraceptives. An over 5-fold increased odds of meningioma was observed in women taking medroxyprogesterone contraceptives, while for women using other hormonal contraceptives, the odds of meningioma did not differ statistically from unexposed unless receiving combination therapies that included medroxyprogesterone.

Our findings support the results from a population-based case-control study of meningioma patients identified from the French National health data system when undergoing meningioma surgery.^21^ Although few cases were exposed to medroxyprogesterone, the OR for meningioma was almost identical with our findings, albeit with a wide confidence interval: 5.55; 95% CI, 2.3-13.6. Griffins analyzed a US Insurance Claims database with 556 cerebral meningioma patients exposed to oral medroxyprogesterone and 207 to medroxyprogesterone injection for > 1 year. No association was observed for oral exposure, while for injections an exposure-response relationship was suggested with ORs in the interval of 1.7-3.4 depending on duration of exposure.^20^ The consistency of the findings and high ORs observed in population-based studies are compelling. Moreover, the recurrence rate after meningioma surgery is higher in women taking progestin only contraceptives, compared to combined contraceptives,^34^ lending further support to our findings.

Our observations agree with 2 previous retrospective, interview-based Nordic studies. A non-overlapping Swedish study found an almost 3-fold increased risk of meningioma associated with long-acting hormonal contraceptives including implants, injections, or intrauterine devices,^35^ while a study conducted in Finland found a modest association for “other” hormonal contraceptives (ie, not oral) and progesterone receptor-positive meningiomas.^36^ The effects of medroxyprogesterone injections may have been diluted in both studies, since data on injections were aggregated with data on implants and intrauterine devices, for which we found no associations in our study.

Epidemiological studies on the progestins nomegestrol acetate^37^ and cyproterone acetate^38^^,^^39^ also showed an association with the risk of meningiomas. Moreover, cyproterone acetate related meningiomas formed a distinct subgroup of hormone dependent tumors.^40^ Discontinuation of cyproterone acetate and nomegestrol acetate is recommended as a first step in management of patients with meningiomas; discontinuation is associated with stabilization or tumor shrinkage.^41^^,^^42^ Taken together, observations of an association between meningiomas and different progestins are uniform. A class-effect and mechanistic biological effects are likely. Neoplasia is related to several hallmarks, including sustained proliferation and evasion of destruction of neoplastic cells.^43^ Progestins can exert such biological effects and can stimulate cell proliferation via mechanisms that require the PR, ERK1/2 and JNK pathways.^44^ In addition, progestins are immunosuppressive^45^ and could attenuate anti-tumoral immunity. There is a known association between progestins and breast cancer which is probably associated with their proliferative effect on breast tissue.^46^

To further address mechanistic implications, patterns of exposure and meningioma were analyzed. Our data revealed a strong association with exposure only to medroxyprogesterone injection. Other preparations were not associated with meningioma occurrence. In our study, medroxyprogesterone was delivered as an injection in a depot-preparation to be repeated every 3 months. In agreement with our findings, there was no association between oral medroxyprogesterone and meningiomas in the US.^20^ Importantly, injection treatment comprised long-term systemic exposure at high constant levels and without any cyclic variation (hormone free break of 7 days every month) which may have been important for the biological effect.

In biology, estrogens and progestins display a complex interplay. The biological implications of unopposed medroxyprogesterone versus simultaneous treatment with estrogens for meningioma are unknown. Progestins are also associated with a risk of breast cancer but seem to act in synergy with estrogens.^47^ In contrast, high progesterone levels during pregnancy are conjunct with estrogens and do not associate with meningioma risk.^19^

### Strengths and Limitations

A major limitation of earlier studies on hormonal contraceptives and meningioma occurrence is that the large majority relied on self-reported information,^6^^,^^12-18^^,^^36^^,^^48-51^ which may introduce recall bias. Case-control studies that rely on questionnaire data are also prone to selection bias: such biases could lead to an over- or underestimation of the association. Another limitation of previous studies was that they often investigated only oral contraceptives, while other types of hormonal contraceptives were rarely evaluated separately.^6^^,^^12-18^^,^^36^^,^^48^^,^^49^^,^^51^ Importantly, 2 recent studies utilized registries and objective data of dispensed medication^20^^,^^21^, however, the number of exposed patients was very low in the French population-based study,^21^ while the American study relied on an insurance-based registry without comprehensive population coverage.^20^

The current study is a register-based case-control study nested within the Swedish population based on comprehensive, high-quality population-based registers. Information regarding prescriptions of hormonal contraceptives was obtained from the Swedish National Prescribed Drug Register. Thus, this study is not affected by selection bias and recall bias. We evaluate the association between specific hormonal contraceptives and meningioma risk, based on their composition and mode of delivery. Almost identical results were observed when performing sensitivity analyses with different exposure definitions, in age-stratified analyses, and after excluding women with congenital malformations, any previous cancer diagnosis, or contraindications for hormonal contraceptives. Another strength is the adjustment for a comprehensive set of potential confounders. In addition, our results were based on a higher number of exposed cases than the previous population-based study,^21^ giving more statistically precise effect estimates.

The main limitation of the current study is that information regarding hormonal contraceptives was available only from July 2005. Therefore, women who had no prescriptions of hormonal contraceptives after July 2005 but had been prescribed hormonal contraceptives before July 2005 are incorrectly classified as unexposed: however, this non-differential exposure misclassification would lead to a dilution of the association and therefore cannot explain the increased odds of meningioma associated with the use of medroxyprogesterone contraceptives. Another limitation is that we could not evaluate whether long-term use of hormonal contraceptives was associated with a further increased risk of meningioma. However, of the 186 women with meningioma who received medroxyprogesterone, only 18 had 3 or fewer prescriptions at least 1 year before the date of meningioma diagnoses and half of the women received at least 15 injections of medroxyprogesterone, meaning that they have received these injections for at least 45 months. In a post hoc analysis we found that women who were prescribed contraceptives containing medroxyprogesterone had a different medical history compared to women who were not taking any hormonal contraceptives. The differential comorbidities comprised diseases of the digestive and musculoskeletal systems and did not include conditions associated with meningioma risk. It is thus unlikely that confounding by indication could explain the strong association that we observed between medroxyprogesterone contraceptives and meningioma occurrence.

### Conclusion

In this large register-based case-control study we found a strong association between prescription of medroxyprogesterone contraceptives and meningioma occurrence: only weak associations were observed for other hormonal contraceptives. However, the absolute risk of developing a meningioma remains low: the 5-fold increased odds observed in our study correspond to 2 additional cases of meningioma per 10 000 women exposed to medroxyprogesterone every year. Our findings agree with recent observations of a progestin association with meningiomas and in particularly strengthen the earlier observations regarding medroxyprogesterone injections and meningioma risk.

## Supplementary Material

noaf228_Supplementary_Data

## Data Availability

The data are pseudonymized individual-level sensitive data from Swedish registers and cannot be shared according to current legislation for data protection. The data can be requested from the respective registry holders, the Swedish National Board of Health and Welfare and Statistics Sweden, after approval by the Swedish Ethical Review Authority.

## Keywords

meningioma | hormonal contraception | epidemiology | women | registries

## References

[1] Baldi I , Engelhardt J, Bonnet C, et al Epidemiology of meningiomas. Neurochirurgie. 2018;64:5–14. 25249493 10.1016/j.neuchi.2014.05.006

[2] Claus EB , Bondy ML, Schildkraut JM, Wiemels JL, Wrensch M, Black PM. Epidemiology of intracranial meningioma. Neurosurgery. 2005;57:1088–1095. 16331155 10.1227/01.neu.0000188281.91351.b9

[3] Price M , Ballard C, Benedetti J, et al CBTRUS statistical report: primary brain and other central nervous system tumors diagnosed in the United States in 2017-2021. Neuro Oncol. 2024;26:vi1–vi85. 39371035 10.1093/neuonc/noae145 PMC11456825

[4] Du Z , Santagata S. Uncovering the links between systemic hormones and oncogenic signaling in the pathogenesis of meningioma. Ann Oncol. 2018;29:537–540. 29346520 10.1093/annonc/mdy010

[5] Bondy M , Ligon BL. Epidemiology and etiology of intracranial meningiomas: a review. J Neurooncol. 1996;29:197–205. 8858525 10.1007/BF00165649

[6] Claus EB , Black PM, Bondy ML, et al Exogenous hormone use and meningioma risk: what do we tell our patients? Cancer. 2007;110:471–476. 17580362 10.1002/cncr.22783

[7] Rao G , Giordano SH, Liu J, McCutcheon IE. The association of breast cancer and meningioma in men and women. Neurosurgery. 2009;65:483–489; discussion 489. 19687693 10.1227/01.NEU.0000350876.91495.E0

[8] Roelvink NC , Kamphorst W, van Alphen HA, Rao BR. Pregnancy-related primary brain and spinal tumors. Arch Neurol. 1987;44:209–215. 3545159 10.1001/archneur.1987.00520140069020

[9] Agopiantz M , Carnot M, Denis C, Martin E, Gauchotte G. Hormone receptor expression in meningiomas: a systematic review. Cancers (Basel). 2023;15:980. 36765937 10.3390/cancers15030980 PMC9913299

[10] Baxter DS , Orrego A, Rosenfeld JV, Mathiesen T. An audit of immunohistochemical marker patterns in meningioma. J Clin Neurosci. 2014;21:421–426. 24231566 10.1016/j.jocn.2013.06.008

[11] Korhonen K , Salminen T, Raitanen J, Auvinen A, Isola J, Haapasalo H. Female predominance in meningiomas can not be explained by differences in progesterone, estrogen, or androgen receptor expression. J Neurooncol. 2006;80:1–7. 16703453 10.1007/s11060-006-9146-9

[12] Claus EB , Calvocoressi L, Bondy ML, Wrensch M, Wiemels JL, Schildkraut JM. Exogenous hormone use, reproductive factors, and risk of intracranial meningioma in females. J Neurosurg. 2013;118:649–656. 23101448 10.3171/2012.9.JNS12811 PMC3756881

[13] Custer B , Longstreth WT Jr, Phillips LE, Koepsell TD, Van Belle G.­ Hormonal exposures and the risk of intracranial meningioma in women: a population-based case-control study. BMC Cancer. 2006;6:152. 16759391 10.1186/1471-2407-6-152 PMC1524800

[14] Michaud DS , Gallo V, Schlehofer B, et al Reproductive factors and exogenous hormone use in relation to risk of glioma and meningioma in a large European cohort study. Cancer Epidemiol Biomarkers Prev. 2010;19:2562–2569. 20802020 10.1158/1055-9965.EPI-10-0447 PMC2990203

[15] Muskens IS , Wu AH, Porcel J, et al Body mass index, comorbidities, and hormonal factors in relation to meningioma in an ethnically diverse population: the multiethnic cohort. Neuro Oncol. 2019;21:498–507. 30615143 10.1093/neuonc/noz005 PMC6422634

[16] Cea-Soriano L , Blenk T, Wallander MA, Rodriguez LA. Hormonal therapies and meningioma: is there a link? Cancer Epidemiol. 2012;36:198–205. 21943794 10.1016/j.canep.2011.08.003

[17] Jhawar BS , Fuchs CS, Colditz GA, Stampfer MJ. Sex steroid hormone exposures and risk for meningioma. J Neurosurg. 2003;99:848–853. 14609164 10.3171/jns.2003.99.5.0848

[18] Lee E , Grutsch J, Persky V, Glick R, Mendes J, Davis F. Association of meningioma with reproductive factors. Int J Cancer. 2006;119:1152–1157. 16570277 10.1002/ijc.21950

[19] Pettersson-Segerlind J , Mathiesen T, Elmi-Terander A, et al The risk of developing a meningioma during and after pregnancy. Sci Rep. 2021;11:9153. 33911184 10.1038/s41598-021-88742-2 PMC8080659

[20] Griffin RL. The association between medroxyprogesterone acetate exposure and meningioma. Cancers (Basel). 2024;16:3362. 39409982 10.3390/cancers16193362 PMC11482550

[21] Roland N , Neumann A, Hoisnard L, et al Use of progestogens and the risk of intracranial meningioma: national case-control study. BMJ. 2024;384:e078078. 38537944 10.1136/bmj-2023-078078 PMC10966896

[22] European Medicines Agency Pharmacovigilance Risk Assessment Committee (PRAC). PRAC recommendations on signals. EMA/PRAC/373353/2024. Accessed January 10, 2025. https://www.ema.europa.eu/en/documents/prac-recommendation/prac-recommendations-signals-adopted-2-5-september-2024-prac-meeting_en.pdf

[23] United Nations Population Division. Contraceptive use by method 2019: data booklet. Accessed March 20, 2025. https://digitallibrary.un.org/record/3849735? v=pdf

[24] Ludvigsson JF , Otterblad-Olausson P, Pettersson BU, Ekbom A. The Swedish personal identity number: possibilities and pitfalls in healthcare and medical research. Eur J Epidemiol. 2009;24:659–667. 19504049 10.1007/s10654-009-9350-y PMC2773709

[25] Barlow L , Westergren K, Holmberg L, Talback M. The completeness of the Swedish cancer register: a sample survey for year 1998. Acta Oncol. 2009;48:27–33. 18767000 10.1080/02841860802247664

[26] Wettermark B , Hammar N, Fored CM, et al The new Swedish prescribed drug register--opportunities for pharmacoepidemiological research and experience from the first six months. Pharmacoepidemiol Drug Saf. 2007;16:726–735. 16897791 10.1002/pds.1294

[27] Ludvigsson JF , Andersson E, Ekbom A, et al External review and validation of the Swedish national inpatient register. BMC Public Health. 2011;11:450. 21658213 10.1186/1471-2458-11-450 PMC3142234

[28] Cnattingius S , Kallen K, Sandstrom A, et al The Swedish medical birth register during five decades: documentation of the content and quality of the register. Eur J Epidemiol. 2023;38:109–120. 36595114 10.1007/s10654-022-00947-5 PMC9867659

[29] World Health Organization. Medical Eligibility Criteria for Contraceptive Use. 4th ed: World Health Organization; 2010.

[30] Ludvigsson JF , Svedberg P, Olen O, Bruze G, Neovius M. The longitudinal integrated database for health insurance and labour market studies (LISA) and its use in medical research. Eur J Epidemiol. 2019;34:423–437. 30929112 10.1007/s10654-019-00511-8 PMC6451717

[31] Ekbom A. The Swedish multi-generation register. Methods Mol Biol. 2011;675:215–220. 20949391 10.1007/978-1-59745-423-0_10

[32] Ludvigsson JF , Almqvist C, Bonamy AK, et al Registers of the swedish total population and their use in medical research. Eur J Epidemiol. 2016;31:125–136. 26769609 10.1007/s10654-016-0117-y

[33] Rothman KJ , Greenland S, Lash TL. Modern Epidemiology. Third ed. Philadelphia, PA, USA: Lippincott Williams & Wilkins; 2008.

[34] Harland TA , Freeman JL, Davern M, et al Progesterone-only contraception is associated with a shorter progression-free survival in premenopausal women with WHO grade I meningioma. J Neurooncol. 2018;136:327–333. 29081037 10.1007/s11060-017-2656-9

[35] Wigertz A , Lonn S, Mathiesen T, et al; Swedish Interphone Study Group. Risk of brain tumors associated with exposure to exogenous female sex hormones. Am J Epidemiol. 2006;164:629–636. 16835295 10.1093/aje/kwj254

[36] Korhonen K , Raitanen J, Isola J, Haapasalo H, Salminen T, Auvinen A. Exogenous sex hormone use and risk of meningioma: a population-based case-control study in Finland. Cancer Causes Control. 2010;21:2149–2156. 20730482 10.1007/s10552-010-9634-2

[37] Nguyen P , Roland N, Neumann A, et al Prolonged use of nomegestrol acetate and risk of intracranial meningioma: a population-based cohort study. Lancet Reg Health Eur. 2024;42:100928. 38800110 10.1016/j.lanepe.2024.100928 PMC11127190

[38] Weill A , Cadier B, Nguyen P. J C. Exposition prolongée à de fortes doses d’acétate de cyprotérone et risque de méningiome chez la femme. 2019. Accessed September 9, 2020. https://www.ansm.sante.fr/var/ansm_site/storage/original/application/b632fbd0387cd9e80a8312469ed52d2a.pdf

[39] Weill A , Nguyen P, Labidi M, et al Use of high dose cyproterone acetate and risk of intracranial meningioma in women: cohort study. BMJ. 2021;372:n37. 33536184 10.1136/bmj.n37

[40] Champeaux-Depond C , Weller J, Froelich S, Sartor A. Cyproterone acetate and meningioma: a nationwide-wide population based study. J Neurooncol. 2021;151:331–338. 33394263 10.1007/s11060-020-03672-9

[41] Passeri T , Champagne PO, Bernat AL, et al Spontaneous regression of meningiomas after interruption of nomegestrol acetate: a series of three patients. Acta Neurochir (Wien). 2019;161:761–765. 30783806 10.1007/s00701-019-03848-x

[42] Voormolen EHJ , Champagne PO, Roca E, et al Intracranial meningiomas decrease in volume on magnetic resonance imaging after discontinuing progestin. Neurosurgery. 2021;89:308–314. 34166514 10.1093/neuros/nyab175

[43] Hanahan D , Weinberg RA. Hallmarks of cancer: the next generation. Cell. 2011;144:646–674. 21376230 10.1016/j.cell.2011.02.013

[44] Louw-Du Toit R , Simons M, Africander D. Progestins and breast cancer hallmarks: the role of the ERK1/2 and JNK pathways in estrogen receptor positive breast cancer cells. J Steroid Biochem Mol Biol. 2024;237:106440. 38048919 10.1016/j.jsbmb.2023.106440

[45] Zwahlen M , Stute P. Impact of progesterone on the immune system in women: a systematic literature review. Arch Gynecol Obstet. 2024;309:37–46. 36933040 10.1007/s00404-023-06996-9 PMC10024519

[46] Li CI , Beaber EF, Tang MT, Porter PL, Daling JR, Malone KE. Effect of depo-medroxyprogesterone acetate on breast cancer risk among women 20 to 44 years of age. Cancer Res. 2012;72:2028–2035. 22369929 10.1158/0008-5472.CAN-11-4064 PMC3328650

[47] Opatrny L , Dell’Aniello S, Assouline S, Suissa S. Hormone replacement therapy use and variations in the risk of breast cancer. BJOG. 2008;115:169–175; discussion 175. 18081598 10.1111/j.1471-0528.2007.01520.x

[48] Andersen L , Friis S, Hallas J, Ravn P, Schrøder HD, Gaist D. Hormone replacement therapy increases the risk of cranial meningioma. Eur J Cancer. 2013;49:3303–3310. 23800670 10.1016/j.ejca.2013.05.026

[49] Blitshteyn S , Crook JE, Jaeckle KA. Is there an association between meningioma and hormone replacement therapy? J Clin Oncol. 2008;26:279–282. 18182668 10.1200/JCO.2007.14.2133

[50] Cardis E , Richardson L, Deltour I, et al The INTERPHONE study: design, epidemiological methods, and description of the study population. Eur J Epidemiol. 2007;22:647–664. 17636416 10.1007/s10654-007-9152-z

[51] Korhonen K , Auvinen A, Lyytinen H, Ylikorkala O, Pukkala E. A nationwide cohort study on the incidence of meningioma in women using postmenopausal hormone therapy in Finland. Am J Epidemiol. 2012;175:309–314. 22287638 10.1093/aje/kwr335

