# Supplementary material for: Hormonal contraceptives and the risk of meningioma: A Swedish register-based case-control study
Source: Neuro Oncol. 2025 Oct 7;28(2):552–61. doi: 10.1093/neuonc/noaf228 (PMC12979035; doi:10.1093/neuonc/noaf228)
Supplement: noaf228_Supplementary_Data [file noaf228_supplementary_data.zip › Hormonal contraceptives and meningioma Suppl Tables.docx]

Supplementary Table S1: List of contraindications for hormonal contraceptives used to create a different case-control study to test the robustness of our findings. These contraindications were defined according to the WHO Medical eligibility criteria for contraceptive use, fourth edition

| **Contraindication** | **Category^*^** |
| --- | --- |
| Hypertension and vascular disease | 3/4 |
| Deep venous thrombosis / pulmonary embolism | 4 |
| Known thrombogenic mutations | 4 |
| Current and history of ischaemic heart disease | 4 |
| Stroke | 4 |
| Known hyperlipidaemias | 2/3 |
| Complicated valvular heart disease | 4 |
| Systemic lupus erythematosus | 4 |
| Migraine | 3/4 |
| Breast cancer (current and past) | 3/4 |
| Nephropathy/retinopathy/neuropathy | 3/4 |
| Other vascular disease or diabetes of > 20 years duration | 3/4 |
| Gall bladder disease | 3 |
| History of cholestasis | 2/3 |
| Viral hepatitis (acute or flare) | 2/3/4 |
| Severe cirrhosis | 3/4 |
| Malignant liver tumors or hepatocellular adenoma | 4 |

* Category 2: Generally use the method; Category 3: Use of method not usually recommended unless other more appropriate methods are not available or not acceptable; Category 4: Method not to be used

Supplementary Table S2: Distribution of hormonal contraceptive prescriptions among matched controls (n=21 000)

|  | **Type of hormonal contraceptive** | | | | | | |
| --- | --- | --- | --- | --- | --- | --- | --- |
|  | **Any type** | **Progestins only** | **Medroxy-progesterone** | **Estrogens & progestogens** | **Estrogens & levonorgestrel** | **Subdermal implants** | **Intrauterine devices & vaginal rings** |
|  | n (%) | n (%) | n (%) | n (%) | n (%) | n (%) | n (%) |
| **Total** | 6888 (32.6) | 5532 (26.2) | 863 (4.1) | 2355 (11.2) | 1175 (5.6) | 250 (1.2) | 2490 (11.8) |
| **Age, index date** |  |  |  |  |  |  |  |
| 20-29 | 411 (70.9) | 201 (34.7) | 15 (2.6) | 304 (52.4) | 154 (26.6) | 39 (6.7) | 52 (9.0) |
| 30-39 | 1602 (51.4) | 1105 (35.4) | 115 (3.7) | 868 (27.8) | 415 (13.3) | 74 (2.4) | 450 (14.4) |
| 40-49 | 3438 (36.0) | 2929 (30.6) | 455 (4.8) | 961 (10.1) | 486 (5.1) | 120 (1.3) | 1463 (15.3) |
| 50-60 | 1437 (18.3) | 1297 (16.5) | 278 (3.6) | 222 (2.8) | 120 (1.5) | 17 (0.2) | 572 (7.3) |
| **Parity** |  |  |  |  |  |  |  |
| No children | 1080 (31.4) | 600 (17.5) | 112 (3.3) | 634 (18.5) | 325 (9.5) | 50 (1.5) | 153 (4.5) |
| 1-2 children | 4046 (34.4) | 3357 (28.6) | 481 (4.1) | 1311 (11.2) | 626 (5.3) | 147 (1.3) | 1619 (13.8) |
| ≥ 3 children | 1762 (29.8) | 1575 (26.7) | 270 (4.6) | 410 (6.9) | 224 (3.8) | 53 (0.9) | 765 (12.9) |
| **Marital status** |  |  |  |  |  |  |  |
| Not married | 3582 (34.2) | 2732 (26.1) | 471 (4.5) | 1378 (13.2) | 681 (6.5) | 141 (1.4) | 1130 (10.8) |
| Married | 3306 (31.1) | 2800 (26.4) | 392 (3.7) | 977 (9.2) | 494 (4.7) | 109 (1.0) | 1407 (13.3) |
| **Education** |  |  |  |  |  |  |  |
| ≤ Compulsory | 583 (25.4) | 487 (21.3) | 135 (5.9) | 169 (7.4) | 82 (3.6) | 30 (1.3) | 145 (6.3) |
| Upper secondary | 3240 (32.3) | 2675 (26.7) | 518 (5.2) | 1004 (10.0) | 510 (5.1) | 120 (1.2) | 1151 (11.5) |
| Postsecondary | 3031 (34.9) | 2344 (27.0) | 200 (2.3) | 1172 (13.5) | 578 (6.7) | 97 (1.1) | 1233 (14.2) |
| Missing | 34 (31.8) | 26 (24.3) | 10 (9.4) | 10 (9.4) | 5 (4.7) | 3 (2.8) | 8 (7.5) |
| **Income** (percentiles) |  |  |  |  |  |  |  |
| <25^th^ | 1475 (31.5) | 1170 (25.0) | 222 (4.7) | 544 (11.6) | 276 (5.9) | 79 (1.7) | 445 (9.5) |
| 25^th^ – 50^th^ | 1769 (36.1) | 1429 (29.2) | 229 (4.7) | 632 (12.9) | 313 (6.4) | 76 (1.6) | 621 (12.7) |
| 50^th^ – 75^th^ | 1917 (34.2) | 1548 (27.6) | 236 (4.2) | 628 (11.2) | 309 (5.5) | 51 (0.9) | 755 (13.5) |
| 75^th^ – 100^th^ | 1724 (29.3) | 1382 (23.5) | 176 (3.0) | 551 (9.4) | 277 (4.7) | 44 (0.8) | 714 (12.1) |
| Missing | 3 (20.0) | 3 (20.0) | - | - | - | - | 2 (13.3) |
| **Country of birth** |  |  |  |  |  |  |  |
| Sweden | 5988 (34.6) | 4842 (27.9) | 774 (4.5) | 2002 (11.6) | 1008 (5.8) | 201 (1.2) | 2256 (13.0) |
| Not in Sweden | 900 (23.9) | 690 (18.3) | 89 (2.4) | 353 (9.4) | 167 (4.4) | 49 (1.3) | 281 (7.5) |
| **Previous disease of the circulatory system** |  |  |  |  |  |  |  |
| Yes | 342 (23.8) | 307 (21.4) | 61 (4.2) | 64 (4.5) | 34 (2.4) | 14 (1.0) | 151 (10.5) |
| No | 6546 (33.3) | 5225 (26.6) | 802 (4.1) | 2291 (11.7) | 1141 (5.8) | 236 (1.2) | 2386 (12.1) |
| **Family history of breast cancer** |  |  |  |  |  |  |  |
| Yes | 437 (29.3) | 373 (25.0) | 56 (3.8) | 110 (7.4) | 51 (3.4) | 13 (0.9) | 185 (12.4) |
| No | 6451 (32.9) | 5159 (26.3) | 807 (4.1) | 2245 (11.5) | 1124 (5.7) | 237 (1.2) | 2352 (12.0) |
| **Family history of central nervous system tumors** |  |  |  |  |  |  |  |
| Yes | 128 (33.4) | 106 (27.7) | 15 (3.9) | 49 (12.8) | 22 (5.7) | 5 (1.3) | 49 (12.8) |
| No | 6760 (32.6) | 5426 (26.2) | 848 (4.1) | 2306 (11.1) | 1153 (5.6) | 245 (1.2) | 2488 (12.0) |

Note: Denominators for the percentage calculations can be found in Table 1.

Supplementary Table S3: Association between hormonal contraceptives, prescribed at least one year before the index date, and the occurrence of meningioma after adjusting for body mass index^*^

|  |  |  |
| --- | --- | --- |
|  | ***N* cases/ *N* controls** | **Adjusted OR^†^ (95% CI)** |
| **All hormonal contraceptives** |  |  |
| Unexposed^‡^ | 366/8669 | Ref |
| Exposed | 318/5018 | 1.60 (1.35-1.90) |
| **Progestins only** |  |  |
| Unexposed^‡^ | 366/8669 | Ref |
| Exposed | 294/4258 | 1.74 (1.46-2.07) |
| **Medroxyprogesterone** |  |  |
| Unexposed^‡^ | 366/8669 | ref |
| Exposed | 127/629 | 5.06 (3.94-6.51) |
| **Progestins excluding medroxyprogesterone** |  |  |
| Unexposed^‡^ | 366/8669 | ref |
| Exposed | 167/3617 | 1.13 (0.92-1.39) |
| **Estrogens and progestogens** |  |  |
| Unexposed^‡^ | 366/8669 | ref |
| Exposed | 74/1514 | 1.08 (0.79-1.47) |
| **Estrogens and levonorgestrel** |  |  |
| Unexposed^‡^ | 366/8669 | ref |
| Exposed | 39/751 | 1.23 (0.82-1.84) |
| **Subdermal implants** |  |  |
| Unexposed^‡^ | 366/8669 | ref |
| Exposed | 6/179 | 0.76 (0.31-1.84) |
| **Intrauterine devices and vaginal rings** |  |  |
| Unexposed^‡^ | 366/8669 | ref |
| Exposed | 89/2094 | 1.02 (0.79-1.33) |

* Information regarding body mass index was obtained from the Medical Birth Register. Information on BMI was available for women who had given birth 1982 or later.

† Adjusted for marital status, educational level, income, parity, history of disease of the circulatory system, family history of breast cancer, family history of central nervous system tumors, and body mass index

‡ The reference group in all analyses were women who did not have any prescription of hormonal contraceptives prior to index date

Supplementary Table S4: Medical history of the women included in the study by prescription to hormonal contraceptives

|  | **No prescriptions of hormonal contraceptives** | **Medroxyprogesterone contraceptives** | **Other hormonal contraceptives** |
| --- | --- | --- | --- |
|  | ***N* (%)** | ***N* (%)** | ***N* (%)** |
| Infectious and parasitic diseases | 2444 (17) | 193 (18) | 1333 (20) |
| Diseases of the blood and blood-forming organs | 827 (6) | 65 (6) | 353 (5) |
| Endocrine, nutritional, and metabolic diseases | 1743 (12) | 157 (15) | 665 (10) |
| Mental, behavioral, and neurodevelopmental disorders | 2269 (16) | 201 (19) | 890 (13) |
| Diseases of the nervous system | 2111 (15) | 219 (20) | 910 (14) |
| Diseases of the eye and the ear | 2874 (20) | 265 (24) | 1151 (17) |
| Diseases of the circulatory system | 2203 (15) | 173 (16) | 736 (11) |
| Diseases of the respiratory system | 2820 (20) | 242 (22) | 1489 (22) |
| Diseases of the digestive system | 3836 (27) | 358 (33) | 1734 (26) |
| Diseases of the skin and subcutaneous tissue | 2585 (18) | 202 (19) | 1343 (20) |
| Diseases of the musculoskeletal system and connective tissue | 4436 (31) | 394 (36) | 1904 (29) |
| Diseases of the genitourinary system | 6888 (48) | 516 (48) | 3120 (47) |
| Congenital malformations, deformations, and chromosomal abnormalities | 544 (4) | 70 (6) | 269 (4) |

Supplementary Table S5: Association between hormonal contraceptives, prescribed at least one year before the index date, and the occurrence of meningioma among women who did not have any contraindications for hormonal contraceptives before the index date

|  |  |  | **Age at index date < 45** | | **Age at index date ≥ 45** | |
| --- | --- | --- | --- | --- | --- | --- |
|  | ***N* cases/ *N* controls** | **Adjusted OR^*^ (95% CI)** | ***N c*ases/ *N* controls** | **Adjusted OR^*^ (95% CI)** | ***N c*ases/ *N* controls** | **Adjusted OR^*^ (95% CI)** |
| **All hormonal contraceptives** |  |  |  |  |  |  |
| Unexposed^†^ | 418/10153 | Ref | 113/3012 | ref | 305/7141 | ref |
| Exposed | 363/5524 | 1.77 (1.51-2.07) | 194/3131 | 1.73 (1.34-2.23) | 171/2415 | 1.79 (1.46-2.20) |
| **Progestins only** |  |  |  |  |  |  |
| Unexposed^†^ | 418/10153 | Ref | 113/3012 | ref | 305/7141 | ref |
| Exposed | 315/4421 | 1.91 (1.61-2.25) | 153/2279 | 1.80 (1.38-2.36) | 162/2142 | 1.94 (1.57-2.40) |
| **Medroxyprogesterone** |  |  |  |  |  |  |
| Unexposed^†^ | 418/10153 | Ref | 113/3012 | ref | 305/7141 | ref |
| Exposed | 139/631 | 5.66 (4.49-7.13) | 55/232 | 6.63 (4.37-10.06) | 84/399 | 5.34 (4.02-7.09) |
| **Progestins excluding medroxyprogesterone** |  |  |  |  |  |  |
| Unexposed^†^ | 418/10153 | ref | 113/3012 | ref | 305/7141 | ref |
| Exposed | 176/3781 | 1.17 (0.96-1.42) | 98/2039 | 1.21 (0.89-1.63) | 78/1742 | 1.09 (0.83-1.43) |
| **Estrogens and progestogens** |  |  |  |  |  |  |
| Unexposed^†^ | 418/10153 | Ref | 113/3012 | ref | 305/7141 | ref |
| Exposed | 106/1929 | 1.28 (0.98-1.68) | 84/1455 | 1.40 (1.00-1.96) | 22/484 | 1.08 (0.68-1.72) |
| **Estrogens and levonorgestrel** |  |  |  |  |  |  |
| Unexposed^†^ | 418/10153 | ref | 113/3012 | ref | 305/7141 | ref |
| Exposed | 55/997 | 1.26 (0.90-1.77) | 45/715 | 1.42 (0.94-2.15) | 10/282 | 0.90 (0.46-1.75) |
| **Subdermal implants** |  |  |  |  |  |  |
| Unexposed^†^ | 418/10153 | ref | ‡ | - | ‡ | - |
| Exposed | 9/190 | 1.26 (0.60-2.62) |  |  |  |  |
| **Intrauterine devices and vaginal rings** |  |  |  |  |  |  |
| Unexposed^†^ | 418/10153 | ref | 113/3012 | ref | 305/7141 | ref |
| Exposed | 85/2060 | 1.03 (0.79-1.33) | 43/1045 | 0.96 (0.65-1.43) | 42/1015 | 1.02 (0.72-1.44) |

* Adjusted for marital status, educational level, income, parity, history of diseases of the circulatory system, and family history of breast cancer and central nervous system tumors.

† The reference group in all analyses were women who did not have any prescription of hormonal contraceptives prior to index date

‡ Less than 5 observations in one subgroup
